# LVAD Pump Flow Does Not Adequately Increase With Exercise

**DOI:** 10.1111/aor.13349

**Published:** 2018-11-18

**Authors:** Christoph Gross, Christiane Marko, Johann Mikl, Johann Altenberger, Thomas Schlöglhofer, Heinrich Schima, Daniel Zimpfer, Francesco Moscato

**Affiliations:** ^1^ Center for Medical Physics and Biomedical Engineering Medical University of Vienna Vienna Austria; ^2^ Ludwig‐Boltzmann‐Cluster for Cardiovascular Research Vienna Austria; ^3^ PVA Center for Ambulatory Rehabilitation Vienna Vienna Austria; ^4^ Rehabilitation Center Felbring Felbring Austria; ^5^ Rehabilitation Center Großgmain Großgmain Austria; ^6^ Paracelsus Medical University Salzburg Austria; ^7^ Department of Cardiac Surgery Medical University of Vienna Vienna Austria

**Keywords:** Left ventricular assist device, Mechanical circulatory support, Exercise, Cardiac response, Cardiac rehabilitation

## Abstract

Left ventricular assist devices (LVADs) restore cardiovascular circulatory demand at rest with a spontaneous increase in pump flow to exercise. The relevant contribution of cardiac output provided by the LVAD and ejected through the aortic valve for exercises of different intensities has been barely investigated in patients. The hypothesis of this study was that different responses in continuous recorded pump parameters occur for maximal and submaximal intensity exercises and that the pump flow change has an impact on the oxygen uptake at peak exercise (pVO_2_). Cardiac and pump parameters such as LVAD flow rate (*Q*_LVAD_), heart rate (HR), and aortic valve (AV) opening were analyzed from continuously recorded LVAD data during physical exercises of maximal (bicycle ergometer test) and submaximal intensities (6‐min walk test and regular trainings). During all exercise sessions, the LVAD speed was kept constant. Cardiac and pump parameter responses of 16 patients for maximal and submaximal intensity exercises were similar for *Q*_LVAD_: +0.89 ± 0.52 versus +0.59 ± 0.38 L/min (*P* = 0.07) and different for HR: +20.4 ± 15.4 versus +7.7 ± 5.8 bpm (*P *< 0.0001) and AV‐opening with 71% versus 23% of patients (*P* < 0.0001). Multi‐regression analysis with pVO_2_ (*R* ^2^ = 0.77) showed relation to workload normalized by bodyweight (*P* = 0.0002), HR response (*P *= 0.001), AV‐opening (*P *= 0.02), and age (*P *= 0.06) whereas the change in *Q*_LVAD_ was irrelevant. Constant speed LVADs provide inadequate support for maximum intensity exercises. AV‐opening and improvements in HR show an important role for higher exercise capacities and reflect exercise intensities. Changes in pump flow do not impact pVO_2_ and are independent of AV‐opening and response in HR. An LVAD speed control may lead to adequate left ventricular support during strenuous physical activities.

Increased support duration with newer generations left ventricular assist devices and availability of donor organs only for selected patients result in a widespread use of left ventricular assist devices (LVAD) 1. Smaller LVAD devices that allow less invasive surgical implantation techniques as well as enhanced strategies to manage blood pressure and anti‐coagulation have been important improvements of LVAD therapy 2.

LVADs increase perfusion, reduce pulmonary pressures, improve quality of life, and submaximal exercise capacity compared to pre LVAD condition 2, 3, 4, 5, 6. Significant improvements in NYHA functional class from NYHA class prior to LVAD implant of IIIb‐IV to I–II after 1 year were reported 7, 8. LVAD patients from our center perform cardiac rehabilitation to restore and improve aerobic work capacity, physical strength, and mobility after LVAD implantation 9. Exercise training for LVAD patients is safe and effective 10, 11, 12 with significant improvements in exercise capacity and quality of life scores after at least 6 weeks of training 11. However very heterogeneous inter‐individual exercise and functional capacities were reported, which are due to the different severities of heart failure and the presence of other organs’ dysfunction 10, 12.

Maximal physical capacity in patients during LVAD support remains very low. According to the literature 12, 13 oxygen uptake at peak exercise (pVO_2_) in LVAD recipients only reaches 40–50% of the predicted pVO_2_. The limited exercise capacities observed in most patients are related to the pathophysiological changes of the cardiovascular, neurologic, respiratory, and musculoskeletal system 12. These changes result in reduced cardiac output and pVO_2_ 14, 15, 16, 17, 18, 19. Factors that could influence cardiac output and thus aerobic capacity are left and right ventricular function, chronotropic response, pulmonary functional capacity, endothelial function, and anemia 12, 13.

Most LVADs used nowadays are axial or centrifugal rotary blood pumps operating at a constant speed 20. These LVADs are sensitive to preload and afterload, because the intrinsic dependency of the pump flow from the pump’s head pressure (= aortic pressure–left ventricular pressure) 21. In previous studies, it has been shown that LVAD pump flow increases with exercise 15, 18. However, the level of LVAD flow increase to various submaximal exercises remained unclear.

Due to the complex interaction of the native left ventricle (LV), the LVAD, and the overall circulatory system, it is not yet well known if and to which extent the LVAD output contributes to maximal and submaximal exercises. In this study, a unique method to continuously monitor patients’ LVAD data from implant onward are used allowing observations of the LVAD output and other related factors during stationary as well as nonstationary exercises.

The aim of this study is to compare pump and cardiac response during physical exercises at maximal and submaximal levels as well as to explore associations of parameters with pVO_2_. Furthermore, exercise related increase in pump flow is investigated together with cardiac parameters during maximum exercise tests. We hypothesized that even at constant speed, the change in pump flow and cardiac response in LVAD patients is higher at maximal compared to submaximal exercise and that the increase in pump flow has an impact on the pVO_2_.

## Patients and Methods

In order to investigate LVAD patients’ exercise responses, LVAD data were monitored during cardiac rehabilitation from the patients within the clinical study for continuous LVAD monitoring. LVAD data together with the obtained exercise parameters were analyzed retrospectively.

### LVAD monitoring

A data recorder has been developed to record the continuous data stream provided by the HVAD (Medtronic Inc., Minneapolis, MN, USA) serial port. The recorder is battery‐powered and small in size (80 × 48 × 28 mm) to fit within the patient’s carriage bag for LVAD peripherals. Pump data were acquired with 50 samples/sec and stored onto a miniSD card. For safety reasons, the data link from the controller is galvanic isolated therefore the recorder cannot interfere with the controller under any circumstances. Previously developed algorithms to analyze the cardiac function were applied on the estimated pump flow 22. For each cardiac cycle, parameters such as mean LVAD output (*Q*_LVAD_), aortic valve (AV) opening 23, 24 and heart rate (HR) in terms of beat‐to‐beat interval 25 were calculated from the LVAD pump flow waveform. Pump data were retrospectively analyzed together with the exercise and testing protocols using Matlab (The Mathworks Inc., Natick, MA, USA).

### Clinical study

In a continuing prospective observational study approved by the Institutional Review Board of the Medical University Vienna (ClinicalTrials.gov identifier: NCT01981642), patient data for LVAD diagnostic purposes were recorded. Patients with an HVAD implanted at the General Hospital in Vienna (Austria) with age ranging from 18 to 70 years, no coagulopathies and ability to cope with LVAD peripherals determined by VAD coordinators gave written consent and were enrolled into the clinical study. For this study, only the patients with recorded LVAD data during cardiac rehabilitation were analyzed.

### Evaluation of LVAD data during exercises

Maximal bicycle exercise tests (MBET) consist of cardiopulmonary stress tests and bicycle stress echocardiography. Submaximal intensity exercises comprise the 6‐min walk test (6MWT) and four exercise trainings (interval bicycle ergometer training, walking, mobilization, and strength training for the lower limbs).

Cardiopulmonary stress tests were performed in the upright position on a sitting bicycle ergometer. Bicycle stress echocardiography was performed occasionally with the patient in the semi‐recumbent position. MBETs were performed until subjectively perceived maximum physical capacity symptoms. The incremental increase in workload for MBETs was applied individually based on the patient’s physical performances in the submaximal intensity exercises. The 6‐min walk test was performed indoor usually at the beginning and end of rehabilitation. Based on demographic parameters, expected pVO_2_ were determined by Cooper and expected 6‐min walk distance was calculated with the method by Enright 26.To improve physical capacities, regular exercise training sessions were performed regulated by Borg’s 27 subjectively perceived exertion (for further information on training therapy see 10). To determine LVAD exercise responses, LVAD data were analyzed and combined with the clinical evaluations of MBETs and submaximal intensity exercises.

Responses for MBETs and submaximal intensity exercises were calculated by the difference of baseline values prior to exercise and peak exercise. Data available for multiple medical training sessions and exercise tests were pooled for each patient before calculating the average responses of all patients. Aortic valve status was determined by the beat‐to‐beat calculated aortic valve opening prevalence 23, 24 with AV closed (<20% AV‐opening), intermittent AV‐opening (20–90% AV‐opening), and complete AV‐opening (≥90% AV‐opening).

### Statistical analysis

Measurable responses to exercise were verified for normal distribution with a Kolmogorov–Smirnov test and means  ±  standard deviations (STD) were calculated. To test the differences of response in *Q*_LVAD_ and HR for maximal and submaximal intensity exercises, the Bonferroni–Holm adjusted 2 sample *t*‐test for unequal variances was used. Bonferroni–Holm adjusted Fisher’s exact test was used to test differences in number of patients with complete AV‐opening. For MBETs, the relationship of the *Q*_LVAD_ response with HR response was tested with Pearson correlation. The differences in ∆*Q*_LVAD_ based on AV‐opening during bicycle ergometer stress tests were analyzed with the Bonferroni–Holm adjusted 2 sample *t*‐test for unequal variances. For statistical tests, the significance level of *P* < 0.05 was used. For the cardiopulmonary stress tests, a stepwise regression analysis was performed to estimate the relations of pVO_2_ with parameters associated with exercise capacity such as age, bodyweight, cardiovascular parameters, and muscular performance. The initial model was created with all variables and compared with the explanatory power of models with fewer variables. Predictors with insufficient evidence to reject the null hypothesis that the variable has a zero coefficient were excluded from the model. Backwards elimination of predictor variables was performed for *P *> 0.1.

## Results

### Patient population and cardiac rehabilitations

LVAD data of 16 patients were recorded during cardiac rehabilitation from February 2014 until December 2016. The patient demographics are shown in Table 1. Patients underwent cardiac rehabilitation immediately following hospitalization due to initial LVAD implantation and/or later. In particular 14 of the 16 patients underwent cardiac rehabilitation on postoperative day (POD) 47 ± 22, whereas seven patients performed an additional cardiac rehabilitation on POD 628 ± 327. Average duration for in‐patient cardiac rehabilitation was 35 ± 8 days during which LVAD data were recorded for 84% of days. The average LVAD impeller speed among all patients and during all rehabilitation periods was 2884 ± 221 rpm.

**Table 1:** Patient demographics at LVAD implant

	*n*	Mean ± STD
Patients	16	
Gender (male/female)	14/2 (87/13%)	
Age (years)		57.4 ± 12.8
BMI (kg/m^2^)		27.9 ± 5.1
Intermacs level	1:5 (31%)	
	2:2 (13%)	
	3:5 (31%)	
	4:4 (25%)	
Etiology: CMP (isc. / non‐isc.)	7/9 (44/56%)	
LVAD indication	BTT: 6 (38%)	
	BTC: 5 (31%)	
	BTR: 1 (6%)	
	DT: 4 (25%)	
*Co‐morbidities*		
Diabetes mellitus	5 (31%)	
Pulmonary hypertension	4 (25%)	
Arterial hypertension	7 (44%)	
Atrial fibrillation	3 (19%)	
ICD	11 (69%)	
Renal Insufficiency	4 (25%)	
COPD	3 (19%)	

BTT, bridge to transplantation, BTC, bridge to candidacy, BTR, bridge to recovery, DT, destination therapy.

### Comparison of LVAD data during exercise

**Figure 1:**
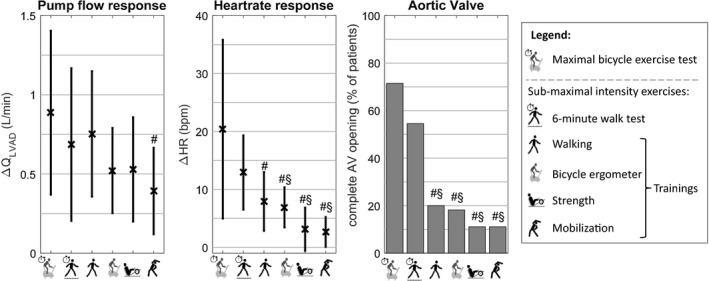
Comparison of physical capacity tests and medical trainings for responses in *Q*_LVAD_, HR and AV‐opening at peak exercise. #: statistical difference with maximum bicycle exercise test, §: statistical difference with 6‐minute walk test.

LVAD data during MBETs (*n* = 24) and submaximal intensity exercises comprising 6MWT (*n* = 16), bicycle ergometer training (*n* = 100), walking training (*n* = 137), strength training (*n* = 71), and mobilization training (*n* = 134) were analyzed. Figure 1 shows the comparison of the average responses for maximal and submaximal intensity exercises. LVAD output increased by +0.89 ± 0.52 L/min during MBETs, +0.69 ± 0.48 L/min during 6MWT and on average by +0.55 ± 0.33 L/min during all training sessions. The increase in HR was +20.4 ± 15.4 bpm during MBETs, +12.9 ± 6.4 bpm during 6MWT and +5.3 ± 4.3 bpm during training sessions. Complete AV‐opening occurred in 71% of patients during MBETs, in 55% during 6MWT and in 15 ± 4.6% during training sessions. The achieved 6‐min walk distance was 316 ± 80 m and corresponds to 52 ± 9% of the expected distance.

For MBETs an average peak workload of 0.61 ± 0.34 W/kg was performed within 7.8 ± 1.9 min. Complete AV‐opening at peak exercise occurred in 10 patients, in two patients the AV remained closed and the remaining two patients showed intermittent AV‐opening. No correlation (*r* = 0.11, *P* = 0.7) between response in *Q*_LVAD_ and HR existed. Similar *Q*_LVAD_ response for complete AV‐opening and intermittent AV‐opening together with AV closed (+0.87 ± 0.52 vs. +0.94 ± 0.58 L/min, *P* = 0.8) were observed.

### Cardiopulmonary bicycle ergometer stress tests

Twelve of the 16 patients underwent a total of 18 cardiopulmonary bicycle stress tests. The predictors of the regression model were peak workload normalized by bodyweight (*P* = 0.0002), ∆HR (*P* = 0.001), peak AV‐opening prevalence (*P* = 0.02), and age (*P* = 0.06). From the initial set of predictors considered in the regression model, ∆*Q*_LVAD_ (*P* = 0.8) was not predictive enough. The multilinear regression model explained (*R* ^2^) 77% of the variation in pVO_2_ (*P* = 0.0001) and is expressed in Eq. 1. Clinical data combined with LVAD data from the cardiopulmonary stress tests are shown in Table 2.

(1) $${\text{pVO}}_{2}=9.67+5.54*\text{Workload/Bodyweight}-0.09*\Delta \text{HR}+0.03*{\text{AV}}_{\text{opening}\;\text{prevalence}}-0.06*\text{Age}$$

**Table 2:** Peak exercise responses for cardiopulmonary bicycle stress tests

	Mean ± STD
Exercise duration (min)	8.0 ± 1.6
pVO_2_ (mL/kg/min)	9.9 ± 2.3
Expected pVO_2_ (%)	35 ± 2.5%
Respiratory exchange ratio (RER)	1.1 ± 0.1
VE/VCO2	47.8 ± 6.7
Maximum workload (W/kg)	0.55 ± 0.28
∆*Q*_LVAD_ (L/min)	+1.0 ± 0.6 (*P* < 0.0001)
∆Heart rate (bpm)	+16.7 ± 15.1 (*P* = 0.0002)
Aortic valve at peak exercise (% of patients, *n*)	AV closed: 17% (2 of 12)
	Intermittent AV‐opening: 17% (2 of 12)
	Complete AV‐opening: 66% (8 of 12)
	(*P *= 0.04)

## Discussion

The main mechanism that leads to an increase in LVAD output at constant speed is a decrease in the pump’s head pressure. The key determinates for a decreased pump head are increasing venous return 28 and LV contractility or reducing afterload, for example, due to vasodilation. These effects occur as a cardiovascular response during exercise depending on exercise intensity and duration 15, 18.

The results from the current study suggest that this increase in pump flow (when an LVAD is driven at constant speed) is moderate and similar for both maximal and submaximal exercises. While the increase in LVAD output in response to low‐intensity exercise seem sufficient, additional improvements to maximal exercise was expected. Indeed, the response of the supported heart (reflected by the ∆HR and AV‐opening) was proportional to the intensity (shown in Fig. 1), whereas the ∆*Q*_LVAD_ remained unchanged. The additional circulatory demand during MBETs therefore must be compensated by the remaining cardiovascular function rather than supported by an increase in LVAD output.

This result underlines the importance of the remaining LV contractility to generate ejection through the AV additional to the pump output. AV‐opening prevalence at peak exercise and not ∆*Q*_LVAD_ was a sufficient predictor of exercise capacity (see Eq. 1). This confirms that the necessary additional cardiac output during peak exercise is mainly generated by the ejection through the AV, as it was also reported in Refs. 15, 18, 29.

Additionally, when the AV opens, a competition between the supported ventricle and the LVAD can occur. The increasing ejection through the AV leads to a reduction of the concomitant increase expected in pump output due to the LVADs’ pump head sensitivity. Increased ejection through the AV results in increased arterial pulse pressure and afterload 15, 18, 30. At peak exercise, preload and afterload together with the ejection through the AV are maximized 15, 16, 17, 18, 19. Depending on the remaining LV contractility during exercise, the increase in ejection through the AV results in increased afterload and leads to attenuation of the increase in LVAD output.

This competing mechanism explained in the previous paragraph, where the ejection through the AV has a relevant concomitant effect on LVAD output is evident in additional results. During MBETs, similar ∆*Q*_LVAD_ for closed and open AVs were obtained despite the existing relationship of AV‐opening and exercise capacity (see Eq. 1). Furthermore, higher exercise capacities were obtained by patients with a better LV function, in this study analyzed by ∆HR and AV‐opening, as reported in Ref. 31. Additionally, heart rate is a potent facilitator of the competing mechanism and no correlation between ∆HR and ∆*Q*_LVAD_ existed, as mentioned in reference 28.

During submaximal trainings, the limited cardiovascular contribution resulted in more patients remaining in full‐support (see Fig. 1) with the whole cardiac output being provided by the LVAD. With increasing exercise intensity increase in preload, inotropic, chronotropic, and vascular response leads to ejection through the AV and reduction of the concomitant increase in *Q*_LVAD_. Such behavior can be seen in Ref. 32 where ∆*Q*_LVAD_ was +1.3 L/min from rest to light exercise, +0.2 L/min from light to moderate exercise, and +0.3 L/min from moderate to heavy exercise. Such dependency of ∆*Q*_LVAD_ on exercise intensity additionally contributes to the significantly higher (*P* = 0.003) expected values for submaximal 6‐min walk distance compared with expected pVO_2_s in this study and mentioned in Ref. 32.

This study shows that LVAD pump output at peak exercise is rather limited and patients with better exercise capacity rely heavily on their remaining cardiovascular function. LVAD speed increase during exercise results in potential benefits independent on the patient’s ability to increase cardiac output through the AV. Patients in full‐support at peak exercise with constant LVAD speed would increase cardiac output and reduce LV preload whereas in partially supported patients a better unloading would allow the supported ventricle to still keep some of its functional reserve. It seems important that the strategy of support depends on the underlying remaining function of the assisted left ventricle and the right ventricle. Therefore, strategies to change pump speed in response to exercise will be different depending on the individual patients’ condition.

Furthermore, the question if current miniaturized LVADs are powerful enough to overtake the whole cardiac output at peak exercise in “less‐sick” patients has to be addressed, since these patients would most likely be in a condition of partial support with their left ventricle engaging in “competition” with the LVAD.

Among the factors contributing to the pVO_2_, those representing the remaining cardiovascular function (∆HR and AV‐opening) and the skeletal muscle contractile and metabolic system (performed workload) dominated rather than pump response in terms of LVAD output (see Eq. 1). For patients remaining in full‐support during exercise, *Q*_LVAD_ reflects cardiac output. In partial‐supported patients, ∆HR and AV‐opening seem to be sufficient parameters representing the cardiac contribution to an increase in cardiac output (see Fig. 1). Thus monitoring such as used in this study allows determining cardiac responses as sensed by the LVAD. The measurement about the contribution of the LV to the overall cardiac output would, of course, provide a more comprehensive picture. Continuous LVAD monitoring could be used to guide training sessions which would require additional investigations. Furthermore, improved monitoring could also reveal pattern of daily activity and thus lead to a better understanding of activity efforts and compliance to outpatient training protocols.

This study has some limitations. As for most observational studies, this work presents results about the associations of LVAD parameters during physical exercise rather than causations. The sample size for this study is too small to draw broader conclusions and only patients within the clinical study were analyzed. The quantification of the flow redistribution between the LV and the LVAD would be very interesting to assess the cooperation of the two pumping systems limiting factors at peak exercise. Cardiac output estimation, for example through rebreathing methods 33 were not available during MBETs which would provide important information. Assumptions on the change in CO were based on surrogates such as AV‐opening and ∆HR. Blood pressure measurements would provide valuable information on the observed ∆*Q*_LVAD_ resulting from the decrease in the pump’s head pressure. Due to the low arterial pulse pressure, pulsatility of LVAD patients most blood pressure measurements were unreliable, especially at rest 34.

## Conclusion

For near‐maximal exercise intensities, patients rely heavily on their remaining left ventricular function with left ventricular assist devices providing support sufficiently, at best, for only low‐intensity exercises. LVAD output response is indeed similar for high‐ and low‐intensity exercises. Parameters representing the cardiovascular function such as ∆HR and AV‐opening contribute to pVO_2_s instead of ∆*Q*_LVAD_. Thus indicating greater contribution of the heart than the LVAD to cardiac output at peak exercise compared to rest. An appropriate LVAD speed control may provide optimized pump output at peak exercise and increase exercise capacity of LVAD patients.

## Authors Contributions

FM and HS developed the concept and design, CG, FM, and TS performed the statistical analysis, all authors contributed to the interpretation of data, funding was secured by FM, HS, DZ, CM, JM and JA, CG drafted the article, CG and TS collected the data, all authors performed critical revision of the article and approved the final version.

## Conflict of interest

The authors have declared no conflicts of interest for this article.

## Funding information

This study was supported by the Austrian Science Fund (FWF): KLI357.
